# Supplementary material for: Mechanisms governing the pioneering and redistribution capabilities of the non-classical pioneer PU.1
Source: Nat Commun. 2020 Jan 21;11:402. doi: 10.1038/s41467-019-13960-2 (PMC6972792; doi:10.1038/s41467-019-13960-2)
Supplement: Supplementary file 7 — Source data [file 41467_2019_13960_MOESM7_ESM.zip › Source_Data/FigureS3/FigureS3H_MotifScanOutput/homerResults/motif26.similar.html]

motif26

## Information for motif26

T
C
G
A
C
G
A
T
T
A
C
G
C
G
T
A
A
T
C
G
A
C
G
T
T
G
A
C
T
C
G
A
A
C
G
T
G
C
T
A
A
G
T
C
  
Reverse Opposite:  

T
C
A
G
C
G
A
T
T
G
C
A
A
G
C
T
A
C
T
G
G
T
C
A
T
A
G
C
G
C
A
T
A
T
G
C
G
C
T
A
A
G
C
T
  

|  |  |
| --- | --- |
| p-value: | 1e-31 |
| log p-value: | -7.231e+01 |
| Information Content per bp: | 1.631 |
| Number of Target Sequences with motif | 293.0 |
| Percentage of Target Sequences with motif | 7.58% |
| Number of Background Sequences with motif | 1520.0 |
| Percentage of Background Sequences with motif | 3.39% |
| Average Position of motif in Targets | 254.9 +/- 231.0bp |
| Average Position of motif in Background | 204.2 +/- 136.8bp |
| Strand Bias (log2 ratio + to - strand density) | -0.0 |
| Multiplicity (# of sites on avg that occur together) | 1.04 |
| Motif File: | file (matrix) reverse opposite |

### Similar de novo motifs found

|  |  |  |  |  |  |  |  |
| --- | --- | --- | --- | --- | --- | --- | --- |
| Rank | Match Score | Redundant Motif | P-value | log P-value | % of Targets | % of Background | Motif file |
| 1 | 0.945 | G C A T C T A G G C T A A C T G A C G T A G T C C T G A C A G T G C T A | 1e-29 | -67.896574 | 9.29% | 4.69% | motif file (matrix) |
| 2 | 0.968 | A C G T A C T G C G T A A T C G A C G T A G T C C T G A A G C T | 1e-28 | -66.074059 | 12.34% | 7.02% | motif file (matrix) |
| 3 | 0.946 | C T A G T G C A G A C T A C T G G T C A T A C G C G A T G T A C C G T A A G C T | 1e-27 | -64.149425 | 8.07% | 3.93% | motif file (matrix) |
| 4 | 0.906 | G C T A C A T G A C G T G T C A A C G T A T C G G T C A A T G C G C A T A G T C G C T A A C G T | 1e-21 | -48.957283 | 6.21% | 3.04% | motif file (matrix) |
